# Stiffness characteristics of the lower extremities in Women’s Chinese Basketball Association competition athletes: normative values and position differences

**DOI:** 10.3389/fbioe.2025.1527730

**Published:** 2025-02-06

**Authors:** Dan Wang, Zhihai Wang, Mingming Yang, Kaiyuan Qu, Xinyu Mao, Xin Yang, Zhiye Zhang, Che Shao, Eamonn Delahunt, Wenxuan Fang

**Affiliations:** ^1^ School of Athletic Performance, Shanghai University of Sport, Shanghai, China; ^2^ School of Acupuncture and Massage, Health Preservation and Rehabilitation, Nanjing University of Chinese Medicine, Nanjing, China; ^3^ School of Public Health, Physiotherapy and Sports Science, University College Dublin, Dublin, Ireland; ^4^ Institute for Sport and Health, University College Dublin, Dublin, Ireland

**Keywords:** athletic injuries, athletic performance, biomechanical phenomena, women’s basketball, stiffness

## Abstract

**Purpose:**

Higher stiffenss is expected to augment performance by increasing the utilisation of elastic energy. However, excessive lower extremity stiffness increases the risk of bony injuries; while insufficient stiffness is associated with a higher likelihood of soft tissue injuries. Thus, there might be an ‘optimal’ stiffness value or range that allows for maximising athletic performance while simultaneously minimising risk of sports injury. Basketball players can be classified by position as centres, guards and forwards, with each position characterised by specific injury risks and exercise patterns. Therefore, this study aims to establish normative data for lower extremity stiffness characteristics of players in the Women’s Chinese Basketball Association (WCBA) and compare the characteristics based on position.

**Methods:**

A total of 124 WCBA athletes (over 70% of the WCBA teams) were recruited for this study, including 63 forwards, 22 centres and 39 guards. Stiffness was evaluated before and during the 2020–2021 WCBA season, which was averaged for data analysis. Quasi-static stiffness measurements of muscles and tendons were collected via a handheld myometer on seven sites of each leg. Vertical stiffness was also evaluated by OptoGait system.

**Results:**

Descriptive statistics were used to establish the normative values of stiffness for forwards, centres and guards. The Kruskal Wallis test and *post hoc* Bonferroni pairwise comparisons found significant higher stiffness of the left patellar tendon (PT) in guards than centres (p = 0.004) and in guards than forwards (p = 0.012), right PT stiffness in guards than centres (p = 0.016) and in guards than forwards (p = 0.017), mean PT stiffness in guards than centres (p = 0.003) and in guards than forwards (p = 0.008); stiffness of the right soleus (SOL) in guards than forwards (p = 0.033), stiffness of the left biceps femoris (BF) in forwards than centres (p = 0.049) and in guards than centres (p = 0.038); and stiffness of the left vertical stiffness (hopping) in forwards than centres (p = 0.041).

**Conclusion:**

Forwards, centres and guards were characterised by significantly different stiffness values, which could be utilised for improvement of athletic performance and injury prevention.

## 1 Introduction

Basketball is characterised as a sport involving actions such as jumping, landing and rapid change of directions, which are conductive to the onset of injuries and determine the athletes’ final performance (Reina et al., 2020b). Besides, injury prevention is essential for maintaining optimal performance (Garbenyte-Apolinskiene et al., 2019). Lower extremity injuries (ankle and knee) are most common in basketball (Clifton et al., 2018). With the increasing participation of women in sports, including basketball, concerns about the risks of sports injuries and their influence on performance have been raised (Garbenyte-Apolinskiene et al., 2019).

Stiffness pertains to both injury and performance (Butler et al., 2003). It is defined as the relationship between an applied load and the elastic deformation of a biological structure (Ditroilo et al., 2012). When it comes to the human body, stiffness can be described from the level of a single muscle fibre to the entire body, which is modelled as a mass and spring (Farley and Gonzalez, 1996; Butler et al., 2003). Muscular contraction adds complexity to stiffness characteristics as it creates a link between neuromuscular function and the mechanical properties of the structures (Latash and Zatsiorsky, 1993). Stiffness is essential in sports performance and injury prevention. Within a certain range, high levels of lower-limb stiffness have been positively correlated with performance metrics like jump height, sprint speed, and efficient force transfer due to reduced ground contact time and greater force output (Butler, et al., 2003). These adaptations contribute to greater movement economy and faster response in high-intensity sports settings, potentially enhancing an athlete’s velocity and explosiveness (Brazier et al., 2019). However, too much or too little lower extremity stiffness may cause bony injuries (stress fractures and osteoarthritis) or soft tissue injuries (muscle strains and ligament tears) respectively. Therefore, it is speculated that there could be an ‘optimal’ stiffness value or range that allows for maximising athletic performance while simultaneously minimising risk of sports injury. Furthermore, determining the optimal lower-limb stiffness requires consideration of the sport’s specific demands and the athlete’s individual characteristics, such as anthropometrics and neuromuscular function (Butler et al., 2003; Brazier et al., 2019). As early as 2010, Watsford et al. highlighted that coaches can use lower-limb stiffness measurements during preseason and in-season periods as an injury risk screening tool (Watsford et al., 2010). Accordingly, coaches and sport scientists can modify training loads and implement ongoing athlete monitoring, thereby reducing the risk of injury and minimizing lost training days, ultimately enhancing athletes’ preparedness for competition (Millett et al., 2017).

Basketball players can be classified by position as centres, guards and forwards, with each position characterised by specific injury risks and exercise patterns. For example, it has been reported that centres experience the highest rate of injury, followed by guards and forwards (Meeuwisse et al., 2003). This may be because centres often sustain a greater rate of injury during the jump-off due to the high player concentration in their active areas and resulting greater potential for contact (Meeuwisse et al., 2003). Besides, centres tend to execute more jumps than guards and forwards, which could explain their significant involvement in offensive and defensive rebounds (Ben Abdelkrim et al., 2007). In contrast, guards spend more active time in sprints than forwards and centres, as their position plays a critical role in fast transitions from defence to offence, and *vice versa* (Ben Abdelkrim, et al., 2007). On the other hand, forwards are required to actively participate in all phases of the game - offence, defence and transitionse - resulting in more accelerations and heightened workloads compared to other playing positions (Ben Abdelkrim et al., 2007). Given these differences in activity types and demands across positions, it is reasonable to expect variations in stiffness characteristics among basketball players based on their playing positions.

To the best of our knowledge, there has been no research investigating normative values and positional differences in lower extremity stiffness characteristics in elite Chinese female basketball players, despite this information is of importance for injury prevention and athletic performance. The Women’s Chinese Basketball Association (WCBA) is the premier women’s basketball league in China, accommodating professional teams from across the country. Each team comprises outstanding players with domestic or international backgrounds, whilst some players also represent the Chinese national women’s basketball team in international competitions such as the basketball world cup and the Olympic games. The objective of this study was to establish normative data for lower extremity stiffness characteristics in WCBA players and compare the characteristics of forwards, centres and guards. It was hypothesised that athletes in team sports, such as WCBA players, should manifest a different level of stiffness in the lower extremities that can be characterised by measurements in three positions.

## 2 Materials and methods

### 2.1 Participants

One hundred and twenty-four frontline female basketball players from 12 teams (22.4 ± 2.8 years, 180.6 ± 7.8 cm, 73.3 ± 10.5 kg) including 63 forwards (22.3 ± 2.7 years, 182.0 ± 4.7 cm, 73.7 ± 7.7 kg), 22 centres (21.5 ± 2.3 years, 190.3 ± 5.3 cm, 85.2 ± 11.8 kg) and 39 guards (23.0 ± 3.2 years; 172.7 ± 4.9 cm, 66.0 ± 7.0 kg) were recruited in this study. This represented over 70% of the WCBA teams in China.

Inclusion criteria of the participants were: 1) no serious lower extremity injuries or operations within the last 3 months and 2) 18–30 year-old WCBA players.

The study was approved by the Human Research Ethics Committee of Shanghai University of Sports. Registration approval number 102772019RT016. All the participants signed the consent form prior to the commencement of testing.

### 2.2 Measurements

All the measurements were proformed twice, before and during the 2020–2021 WCBA season. This approach aligns with it in the study of Pruyn et al. (2015), who suggest that multiple measurements can enhance the accuracy and consistency of stiffness data. The average value of the two measurements were utilised for the analysis.

#### 2.2.1 Muscle and tendon stiffness measurements

Quasi-static stiffness measurements of muscles and tendons were collected using a handheld MyotonPRO myometer (Myoton AS, Tallinn, Estonia). The myometer has shown high reliability and validity in measuring stiffness characteristics during field testing (Pruyn et al., 2015). Participants were required to stand barefoot and expose their legs in the anatomical position. To maintain consistency of measurements among participants, marks were drawn on the skin in the following seven sites on each leg: lateral gastrocnemius (LatGast), medial gastrocnemius (MedGast), soleus (SOL), achilles tendon (AT), biceps femoris (BF), rectus femoris (RF), and patellar tendon (PT) (Hermens et al., 2000) (see Table 1). The probe of the myometer was perpendicularly lowered onto the muscle belly, and an automatic mechanical impact was delivered to the muscle and tendon (duration of 15 or 12 ms, a force of 0.3–0.4 N, and a local deformation in the order of a few millimetres). Resultant damped natural oscillations were measured using an in-built accelerometer sampling at 3,200 Hz (Pruyn et al., 2015). Five consecutive measurements were taken at each site. The average of the five measurements was used for later analysis.

**TABLE 1:** Measurement sites of muscles and tendons.

Muscles and tendons	Measurement sites
Soleus	2/3 of the line between the medial condylis of the femur to the medial malleolus
Lateral Gastrocnemius	1/3 of the line between the head of the fibula and the heel
Medial Gastrocnemius	the most prominent bulge of the muscle
Rectus Femoris	50% on the line from the anterior spina iliaca superior to the superior part of the patella
Biceps Femoris	50% on the line between the ischial tuberosity and the lateral epicondyle of the tibia
Achilles Tendon	5 cm above the calcaneus
Patellar Tendon	3 cm above the tibial tuberosity

#### 2.2.2 Vertical stiffness (K_vert_) measurement

The K_vert_ was evaluated by a vertical hop test and a drop jump (DJ) test performed with the OptoGait optical detection system (MicroGate Corporation, Bolzano, Italy). The OptoGait system consists of an Alienware M17x laptop (Dell Alienware Corporation, FL, USA) and two 2-m modules. Each module was equipped with light emitting diodes (LEDs) to record DJ movement (they sensed when the foot left and re-entered the system). The measurements included ground contact time (time between initial contact with the ground and toe-off from the ground) and flight time (time between the toe-off from the ground and re-contact with the ground) (Bishop et al., 2019), and the calculation of K_vert_ was based on the equations published in a 2005 study (Morin et al., 2005). All participants practiced the vertical hop test and performed a DJ at least three times prior to the measurements.

##### 2.2.2.1 Vertical hop test

Participants were required to hop sub-maximally at 2.2 Hz in time to a metronome. They were instructed to keep their hands on their hips and lock their knees to reduce knee input. Ten continuous hops were performed for each leg. If the participants hopped outside ±2% of the set frequency, they repeated the hopping test after a 2-min break. K_vert_ was calculated from the average of 10 hops and was utilised for data analysis.

##### 2.2.2.2 Drop jump test

Participants started by standing on top of a stool (40 cm), with their feet positioned shoulder width apart and hands positioned on the hips. After 3 seconds of countdown, the participants dropped directly down from the stool and immediately performed a maximum vertical jump, with both hands on the hips. They were instructed to ‘land and jump immediately as high as possible’. Three successful DJ test trials were obtained for each participant. If participants failed to complete the DJ with the correct technique or lost their balance during landing, those trials were excluded from the calculations. K_vert_ was calculated from the average of the three DJs utilised for data analysis.

### 2.3 Statistical analysis

Descriptive statistics were utilised to analyse the normative values of stiffness. Normal distribution of the data was tested using the Kolmogorov-Smirnov Test. Homogeneity of variances was verified by the Levene’s test. When the assumptions of normality and homogeneity were met, one-way between-groups analysis of variance (ANOVA) was conducted to test differences in variables among three positions (forwards, centres, guards). Otherwise, the Kruskal Wallis test was performed if the assumption of normality was violated; the Bonferroni was conducted for *post hoc* pairwise comparisons. Statistical significance was set at a 95% level of confidence. All statistical analyses were performed using IBM SPSS Statistics for Windows, Version 26.0 (IBM Corp., Armonk, NY, USA).

## 3 Results

The means and standard deviations of the normative values of stiffness at three positions in female basketball athletes are displayed in Table 2.

**TABLE 2:** Normative values of stiffness in forwards, centres and guards among WCBA athletes.

	Forwards (n = 63)			Centres (n = 22)			Guards (n = 39)		
	Left	Right	Mean	Left	Right	Mean	Left	Right	Mean
AT (N/m)	1,647.3 (134.3)	1,614.3 (131.9)	1,620.8 (105.0)	1,674.2 (156.8)	1,600.5 (130.4)	1,637.3 (125.3)	1,678.9 (127.0)	1,642.0 (109.8)	1,660.5 (99.9)
PT (N/m)	676.7 (360.5)	686.2 (350.0)	681.4 (331.9)	604.8 (358.6)	662.8 (434.3)	633.8 (390.7)	836.9 (362.3)	845.6 (383.1)	841.2 (334.2)
Soleus (N/m)	541.6 (88.7)	534.2 (83.7)	537.9 (79.5)	537.9 (76.9)	539.9 (75.7)	538.9 (68.0)	560.8 (90.6)	570.3 (92.4)	565.5 (82.7)
LatGast (N/m)	549.9 (84.7)	550.7 (84.1)	550.3 (78.0)	539.8 (65.9)	554.6 (64.4)	547.2 (59.2)	548.6 (87.8)	568.5 (88.7)	558.5 (79.8)
MedGast (N/m)	447.0 (65.2)	444.0 (65.6)	445.6 (59.8)	476.5 (70.9)	461.8 (56.5)	469.1 (57.4)	463.7 (84.1)	449.1 (68.6)	456.4 (72.5)
RF (N/m)	269.3 (56.6)	274.2 (62.5)	271.8 (57.3)	262.8 (53.0)	262.2 (58.6)	262.5 (52.9)	273.8 (50.2)	282.8 (52.2)	278.3 (49.3)
BF (N/m)	302.5 (47.5)	307.5 (48.4)	305.0 (45.3)	281.3 (51.5)	291.6 (57.3)	286.5 (52.9)	306.7 (51.7)	312.2 (48.7)	309.4 (47.8)
K_vert_ (DJ) (N/m/kg)	130.1 (62.7)			133.4 (55.6)			122.3 (52.2)		
K_vert_ (Hopping) (N/m/kg)	225.3 (29.5)	223.2 (21.7)	223.9 (22.4)	214.1 (15.4)	218.6 (19.6)	216.3 (14.6)	222.7 (16.5)	223.6 (17.5)	223.2 (14.9)

Table note: Values are presented as mean (SD). AT, achilles tendon; PT, patellar tendon, LatGast = Lateral gastrocnemius, MedGast = Medial gastrocnemius, RF, rectus femoris; BF, biceps femoris; DJ, drop jump, K_vert_ = Vertical stiffness.

**FIGURE 1:**
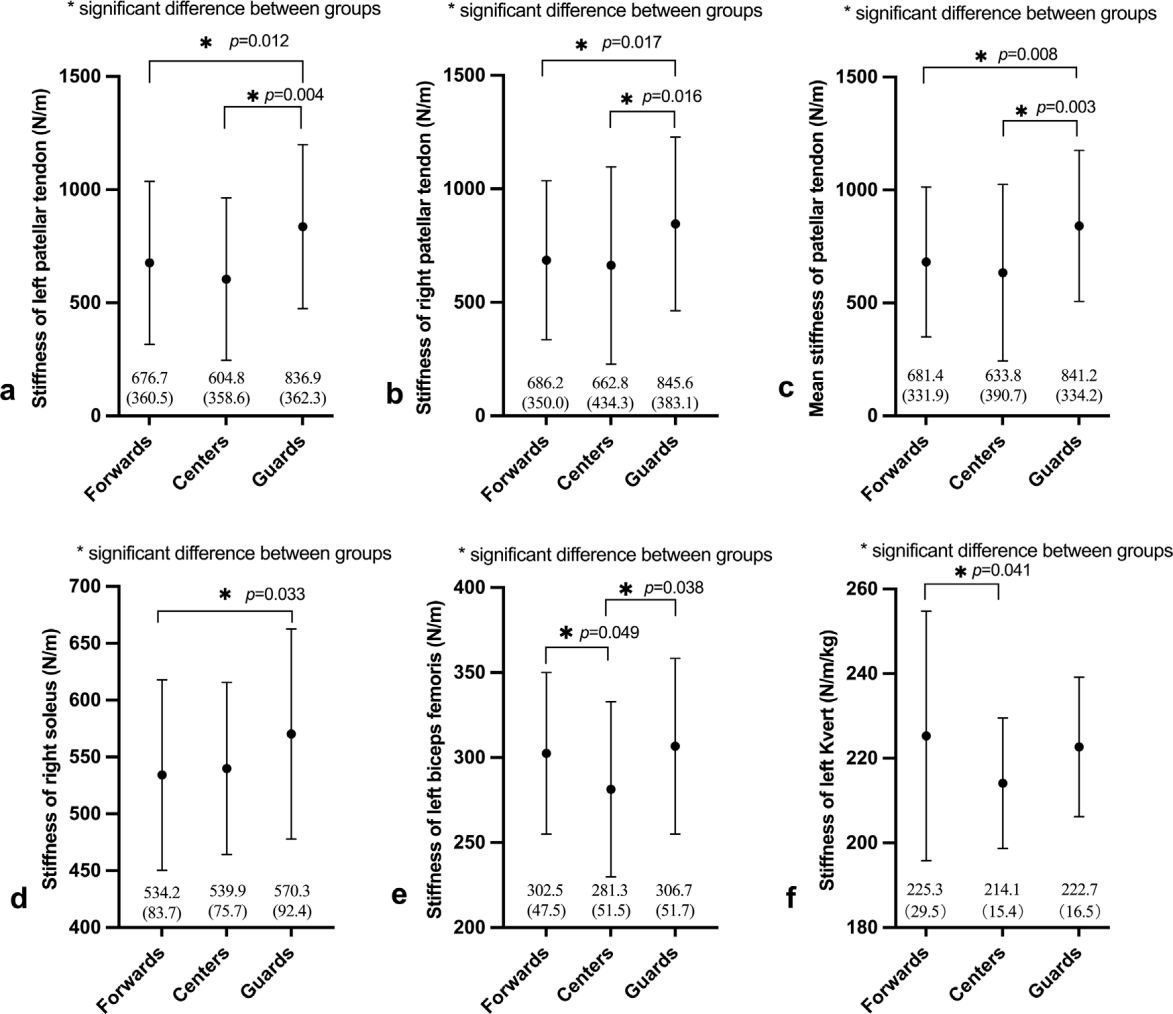
Significant differences of lower extremity stiffness among three positions in WCBA athletes. **(A)** Represents the left patellar tendon stiffness of athletes in the three positions. **(B)** Represents the right patellar tendon stiffness of athletes in the three positions. **(C)** Represents mean stiffness of patellar tendon of athletes in the three positions. **(D)** Represents the right soleus stiffness of athletes in the three positions. **(E)** Represents the left biceps femoris stiffness of athletes in the three positions. **(F)** Represents the left Kvert stiffness of athletes in the three positions. The “*” represents a statistically significant difference between two groups.

**FIGURE 2:**
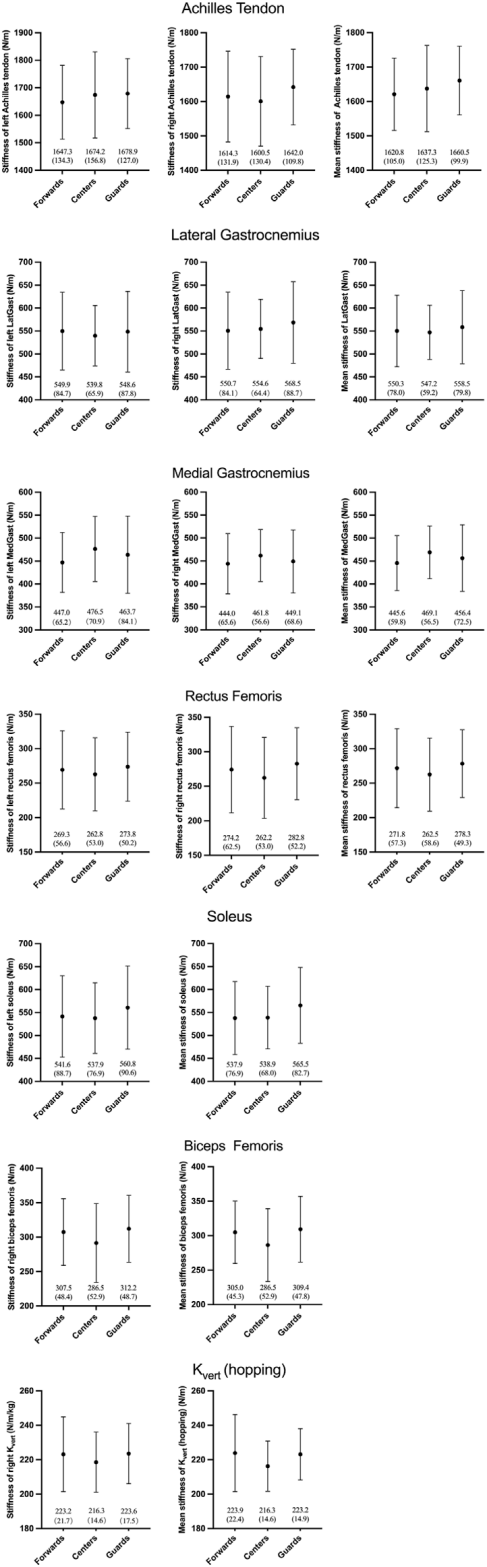
Non-significant differences of lower extremity stiffness among three positions in WCBA athletes.

Since the data about stiffness of the left SOL, left LatGast, mean LatGast, left RF, mean RF, left, right and mean BF, left, right and mean PT, left, right and mean K_vert_ (hopping) were not normally distributed, the Kruskal–Wallis test was conducted. There was a significant difference among the three positions in stiffness of the left PT (X^2^(2) = 12.97, p = 0.002, ε^2^ = 0.07); stiffness of the right PT (X^2^(2) = 10.68, p = 0.005, ε^2^ = 0.05); mean stiffness of PT (X^2^(2) = 13.57, p = 0.001, ε^2^ = 0.07); stiffness of the left BF (X^2^(2) = 7.02, p = 0.03, ε^2^ = 0.07); mean stiffness of RF (X^2^(2) = 6.28, p = 0.023, ε^2^ = 0.06); stiffness of the left K_vert_ (hopping) (X^2^(2) = 5.27, p = 0.043, ε^2^ = 0.05) (small effect size: 0.01 < ε^2^ < 0.08, medium effect size: 0.08 < ε^2^ < 0.26, large effect size: ε^2^ ≥ 0.26) (Vargha and Delaney, 2000). Post-hoc Bonferroni pairwise comparisons found statistically significant higher stiffness measures of the left PT in guards than centres (mean difference = 232.1, p = 0.004) and in guards than forwards (mean difference = 160.2, p = 0.012) (Figure 1A), right PT stiffness in guards than centres (mean difference = 182.8, p = 0.016) and in guards than forwards (mean difference = 159.4, p = 0.017) (Figure 1B), mean PT stiffness in guards than centres (mean difference = 207.4, p = 0.003) and in guards than forwards (mean difference = 159.8, p = 0.008) (Figure 1C); stiffness of the right SOL in guards than forwards (mean difference = 36.1, p = 0.033) (Figure 1D), stiffness of the left BF in forwards than centres (mean difference = 21.2, p = 0.049) and in guards and centres (mean difference = 25.4, p = 0.038) (Figure 1E); and stiffness of the left K_vert_ (hopping) in forwards than centres (mean difference = 11.2, p = 0.041) (Figure 1F). Additionally, refer to Figure 2 for all the non-significant results of the data.

## 4 Discussion

This study established normative data and compared position differences in lower extremity stiffness characteristics among WCBA players. Significant differences were found in left PT stiffness, right PT stiffness and mean PT stiffness between centres and guards, forwards and guards, stiffness of the right SOL between forwards and guards, left BF stiffness between forwards and centres as well as guards and centres, and left K_vert_ (hopping) between forwards and centres.

Tendon and muscle exhibit distinct stiffness properties, which are closely linked to their specific functional roles. Tendons are characterized by higher stiffness, facilitating efficient force transmission and energy storage during dynamic activities (Karamanidis and Arampatzis, 2005). For instance, during stretch-shortening cycles, such as running, the elastic deformation of tendons reduces the mechanical workload on muscles and optimizes force transfer (Butler et al., 2003). In contrast, muscles display relatively lower stiffness, allowing them to actively adjust their length and generate force to accommodate varying movement demands (Karamanidis and Arampatzis, 2005). Additionally, muscle stiffness is modulated by neuromuscular function, providing greater flexibility and control during complex motions (Karamanidis and Arampatzis, 2005). This differentiation underscores the complementary roles of tendon and muscle stiffness: tendon stiffness enhances force transmission and energy efficiency, while muscle stiffness prioritizes adaptability and precise control. Understanding these differences is critical for exploring the interplay between athletic performance and injury mechanisms.

### 4.1 Normative values of lower extremities stiffness in WCBA athletes

The stiffness of the MedGast (Centres - 469.1 ± 57.4 N/m, Guards - 456.4 ± 72.5 N/m, Forwards - 445.6 ± 59.8 N/m vs. 433.7 ± 76.1 N/m), LatGast (Centres - 547.2 ± 59.2 N/m, Guards - 558.5 ± 79.8 N/m, Forwards - 550.3 ± 78.0 N/m vs. 503.3 ± 82.3 N/m) and SOL (Centres - 538.9 ± 68.0 N/m, Guards - 565.5 ± 82.7 N/m, Forwards - 537.9 ± 79.5 N/m vs. 661.8 ± 76.5 N/m) measured in the study were close to those previously reported for netball players (Pruyn et al., 2014). This might be explained by the similar characteristics (some dynamic lower-body movements, including sprinting, bounding and jumping) and the elite athletic level of players for both basketball and netball.

The RF (Centres - 262.5 ± 52.9 N/m, Guards - 278.3 ± 49.3 N/m, Forwards - 271.8 ± 57.3 N/m vs. 349.46 ± 28.53 N/m) and BF (Centres - 286.5 ± 52.9 N/m, Guards - 309.4 ± 47.8 N/m, Forwards - 305.0 ± 45.3 N/m vs. 420.64 ± 38.18 N/m) stiffness measurements for the female basketball players in this study were lower than measurements for elite male soccer players (Kalkhoven and Watsford, 2018). This is possibly due to differences in gender and in sport characteristics. For instance, it has been shown that general stiffness characteristics are significantly higher in men than women (Wang et al., 2016). In addition, soccer players must sprint, change direction, jump, pass and shoot many times during a game, which requires high stiffness levels of RF and BF in soccer players.

The PT stiffness (Centres - 633.8 ± 390.7 N/m, Guards - 841.2 ± 334.2 N/m, Forwards - 681.4 ± 331.9 N/m vs. dominant - 1,045 ± 202 N/m and nondominant - 1,084 ± 193 N/m) reported in this study was lower than that found in a study of breakdancers. This difference may be attributed to continuous stretching and shortening of the RF in breakdancing (Young et al., 2018). During this process, the PT allows the RF to transfer power during the shortening period and release elastic energy during the stretch. Therefore, the PT must adapt to the loads exerted during this type of movement. While the sport of basketball involves a lot of jumping and change of direction (COD) movements, lower PT stiffness may be beneficial as a landing buffer for basketball players, and it functions as a ‘mechanical buffer’ in reducing the rate of force transmission to the muscle to prevent injury. In addition, lower PT stiffness may indicate better stretch-shortening cycle ability and thus improve jump performance and COD.

The AT stiffness (Centres - 1,637.3 ± 125.3 N/m, Guards - 1,660.5 ± 99.9 N/m, Forwards - 1,620.8 ± 105.0 N/m vs. 606.5 ± 84.6 N/m) of the athletes in this study was higher than that reported previously for netball athletes, which could be attributed to a different AT measuring site. AT stiffness was evaluated 5 cm above the calcaneus in this study, whereas Pruyn and colleagues (Pruyn et al., 2014) did not detail the specific site of the measurement and only stated that the Achilles aponeurosis was assessed.

### 4.2 Positional differences in lower extremity stiffness in WCBA athletes

This study found that the left PT, right PT and mean PT stiffness measurements of guards were significantly higher than those of centres and forwards. The PT is an energy-storing structure responsible for transmitting muscle-derived forces to produce joint movement. A stiffer PT better transmits force generated by the muscle to the bone (Cristi-Sánchez et al., 2019) and may enhance sports performance by improving work efficiency. Guards are responsible for transitions from defence to attack in the game (Spiteri et al., 2019). The process involves many CODs (about one time every 1.4–2.8 s) (Spiteri et al., 2019), and a variety of high-intensity slide steps, sprints and dribble runs (Köklü et al., 2011). Within a certain range, greater PT stiffness helps the guards perform better starting and braking during the game, which improves COD and acceleration and allows the guards to better fulfil their responsibilities. Conversely, centres are involved in more low-intensity exercises (walking or standing) than guards. They spend most of the time close to the basket during attack and defence and consequently require more short-distance movement and continuous fast jumping. Forwards are also involved in a great many high-intensity jumping movements; therefore, no significant difference in left PT stiffness was found between centres and forwards. Moreover, when PT stiffness was averaged for both sides, the stiffness values for the guards were still significantly higher than those of centres and forwards (Ben Abdelkrim et al., 2007). Previous studies have shown that PT stiffness plays a crucial role in transmitting muscle-generated forces to the skeletal system, contributing to joint movement and stability (Lee et al., 2017). Lower stiffness allows greater deformation under load, facilitating energy absorption and reducing abrupt stress on the knee joint during high-impact activities like landing (Bravo-Sánchez et al., 2021). Therefore, lower PT stiffness may be more beneficial for the landing buffer among centres and forwards to prevent muscle injury and improve jump performance.

In addition, the AT plays a crucial role in force transmission as well as energy storage and release during jumping and COD movements (Blazevich and Fletcher, 2023). Since all basketball players, regardless of their playing positions, perform a significant amount of jumping or directional changes, this may explain why there were no significant differences in AT stiffness among players in different positions.

This study found that guards’ right SOL stiffness measurements were significantly higher than those of forwards. The soleus is the largest muscle of the triceps, and most of the work/energy in lower extremity was through soleus’s active shortening to lift and accelerate the body during the stance phase of running (Bohm et al., 2021). It also has been shown to have preferential functions in postural control and walking (Koulouris et al., 2007). During a basketball game, guards usually stay near the side-line and are involved more in dribbling (from backcourt to frontcourt) to find offensive opportunities (Zhai et al., 2020), and assure the fast transitions from defence to offence with high-intensity accelerations, thus guards averaged more sprints and spent greater live time in this activity than forwards (Ben Abdelkrim et al., 2007). Evidence proved a positive relationship between higher stiffness and better acceleration capacity (Pruyn et al., 2014). Therefore, higher SOL stiffness contributed to the better acceleration in guards (Ben Abdelkrim et al., 2007). In contrast, forwards are involved in more shots and thus more walking and jumping (Zhai et al., 2020). The lower SOL stiffness may protect muscles against rapid and forceful lengthening damage after a jump for forwards (Konow et al., 2012). Right SOL stiffness of the guards was significantly higher than that of the forwards, which was probably explained by the fact that the right leg was the dominant side for the most basketball players in this study. Basketball players have higher stiffness in the dominant lower extremity than the non-dominant lower extremity (Struzik et al., 2022). This phenomenon might be related to the asymmetrical movements in specific techniques in basketball (e.g., dribble, COD, jump shot, or layups). During running, COD and sprinting, athletes mainly use the dominant side to initiate. As mentioned before, the SOL plays a major role in lifting and accelerating the body during the stance phase of running, hence the activation of SOL stiffness in the right leg of the guards was high. Although the forwards also have short sprints and COD, its overall intensity was lower than that of the guards (Ben Abdelkrim et al., 2007). Therefore, guards have significantly higher right SOL stiffness than forwards.

The results demonstrated that forwards possessed significantly higher left BF stiffness and left K_vert_ when compared with centres. Greater stiffness of the BF means better performance in sprinting, agility and DJ due to the positive relationship between BF stiffness and rate of force development (Kalkhoven and Watsford, 2018). In addition, when the BF is eccentrically contracted, it provides an important buffer function for the body (Ruan and Li, 2010). Higher levels of K_vert_ have generally been associated with superior maximal sprint and jump (Taylor et al., 2017). Forwards are required to participate more in all the game phases, including offence, defence and transitions. Therefore, they may execute more sprints and jumps. Centres jump more than forwards and guards, but centres often work close to the basket with less intensity than forwards (Reina et al., 2020a), while the guards run as much as the forwards (approximately 6,000 m) (Reina et al., 2020a). Therefore, the BF stiffness of guards is also higher than that of centers. Furthermore, it has been reported that centres experience the highest rates of injury, followed by guards and forwards (Donaldson et al., 2003). Lower BF stiffness and K_vert_ may increase the risk of injury for centres. The result also demonstrated that forwards exhibited significantly greater BF stiffness and K_vert_ compared to centers, particularly with notable asymmetry observed on the left side. This asymmetry may be attributed to the right leg serving as the dominant limb for most players in this study, typically used as the preferred leg for explosive movements such as hopping (Shalom et al., 2023). Previous research has documented that asymmetrical lower limb stiffness is a prevalent characteristic among basketball athletes, likely arising from specialized training demands and neuromuscular adaptations (Versic et al., 2021). Given that both forwards and centers engage frequently in sprints and jumps, the dominant leg - typically the right - is more involved in high-intensity movements requiring explosive starts and stops. This pronounced reliance on the dominant side may contribute to the lack of significant stiffness differences in the right leg across player positions (Reina et al., 2020a).

The study found no significant differences in RF and gastrocnemius stiffness among athletes in different positions. Both the RF and gastrocnemius are biarticular muscles. During ground contact, the leg joints (hip, knee, and ankle) typically move synchronously, undergoing a flexion-extension cycle during various functional tasks (Cleland, 1867; Ivanenko et al., 2007). Biarticular muscles are stretched at one joint while being released at another (Van Ingen Schenau, 1989). This unique property helps biarticular muscles maintain joint balance during movement. Additionally, biarticular muscles efficiently transfer mechanical energy between adjacent joints with minimal mechanical work, reducing overall energy expenditure (Wells, 1988; Prilutsky, 2000). During locomotion, muscles like the RF and gastrocnemius transfer mechanical energy from proximal to distal joints in the push-off phase, facilitating joint extension (Prilutsky and Zatsiorsky, 1994). Conversely, during initial contact and early stance, they transfer energy from distal to proximal joints, aiding in energy dissipation. This flexible energy transfer mechanism reduces energy consumption while enhancing movement efficiency and stability. Therefore, the primary roles of the RF and gastrocnemius are to maintain joint balance and enable efficient force transfer between joints. These roles are critical for basketball athletes in all positions, which explains why no positional differences in stiffness were observed among basketball players.

### 4.3 Limitation of this study

The measurements in this study were conducted before and during the season, which made it challenging to include all teams due to their extremely intensive schedules. Consequently, this study focused exclusively on Chinese female basketball athletes. Future research could expand to include male basketball players to provide a broader perspective. Additionally, while we considered the potential impact of the menstrual cycle on lower limb stiffness, the limited testing timeframe and inconsistent findings in the existing literature (Vescovi, 2011) made it impractical to explore this factor in detail. As such, investigating the menstrual cycle’s effect on lower limb stiffness lies beyond the scope of this study.

## 5 Conclusion

This study is the first to report normative data of lower extremity stiffness in WCBA players in the positions of centre, forward and guard. Furthermore, the centres and forwards possessed higher left BF, left K_vert_ stiffness, while the guards were characterised by higher left PT and right PT stiffness, mean PT stiffness and right SOL stiffness. Therefore, centers and forwards should increase BF and K_vert_ stiffness to improve their performance in sprinting and jumping, while guards should aim to increase PT stiffness to optimize acceleration, deceleration, and quick direction changes. Forwards have similar requirements to centers but must also balance flexibility and strength more effectively. In terms of injury prevention, players with too low stiffness, such as centers and forwards, should strengthen muscular strength to mitigate the risks of excessive joint motion and soft tissue injuries and focus on enhancing joint stability to reduce the risk of injuries associated with high-intensity jumping. Conversely, guards with too high stiffness should emphasize improving flexibility to reduce the likelihood of fractures. These findings provide a novel evaluation variable for performance enhancement in female basketball athletes and suggest potential applications in injury prevention, which could be explored in future research.

## Data Availability

The raw data supporting the conclusions of this article will be made available by the authors, without undue reservation.
